# Decreased Expression of AZGP1 Is Associated with Poor Prognosis in Primary Gastric Cancer

**DOI:** 10.1371/journal.pone.0069155

**Published:** 2013-07-23

**Authors:** Chun-yu Huang, Jing-jing Zhao, Lin Lv, Yi-bing Chen, Yuan-fang Li, Shan-shan Jiang, Wei Wang, Ke Pan, Yan Zheng, Bai-wei Zhao, Dan-dan Wang, Yong-ming Chen, Lei Yang, Zhi-wei Zhou, Jian-chuan Xia

**Affiliations:** 1 Department of Endoscopy, Sun Yat-sen University Cancer Center, Guangzhou, P.R. China; 2 State Key Laboratory of Oncology in South China and Department of Experimental Research, Sun Yat-sen University Cancer Center, Guangzhou, P.R. China; 3 Department of Gastric and Pancreatic Surgery, Sun Yat-sen University Cancer Center, Guangzhou, P.R. China; 4 Department of Biotherapy Center, Sun Yat-sen University Cancer Center, Guangzhou, P.R. China; University of Barcelona, Spain

## Abstract

**Background:**

2-Zinc-glycoprotein 1 (AZGP1) is a multidisciplinary protein that participates in many important functions in the human body, including fertilization, immunoregulation and lipid mobilization. Recently, it has been shown that AZGP1 is also involved in carcinogenesis and tumor differentiation. In this study, we investigated the expression levels and prognostic value of AZGP1 in primary gastric cancers.

**Methods and Results:**

We examined the expression of AZGP1 in 35 paired cancerous and matched adjacent noncancerous gastric mucosa tissues by real-time quantitative RT-PCR (qRT-PCR) and western blotting. Furthermore, we analyzed AZGP1 expression in 248 patients who underwent resection procedures between 2005 and 2007 using immunohistochemistry. The relationships between the AZGP1 expression levels, the clinicopathological factors, and patient survival were investigated. AZGP1 expression was significantly reduced at both the mRNA (*P* = 0.023) and protein levels (*P* = 0.019) in tumor tissue samples, compared with expression in matched adjacent non-tumor tissue samples. The immunohistochemical staining data showed that AZGP1 expression was significantly decreased in 52.8% (131/248) of gastric adenocarcinoma cases. Clinicopathological analysis showed that the reduced expression of AZGP1 was significantly correlated with tumor location (*P* = 0.011), histological grade (*P* = 0.005) and T stage (*P* = 0.008). Kaplan–Meier survival curves revealed that the reduced expression of AZGP1 was associated with a poor prognosis in gastric adenocarcinoma patients (*P* = 0.009). Multivariate Cox analysis identified AZGP1 expression was an independent prognostic factor for overall survival of gastric adenocarcinoma patients (HR = 1.681, 95% CI = 1.134–2.494, *P* = 0.011).

**Conclusions:**

Our study suggests that AZGP1 might serve as a candidate tumor suppressor and a potential prognostic biomarker in gastric carcinogenesis.

## Introduction

Gastric cancer is the second most common cause of cancer-related mortality worldwide, with 988,000 new cases and 736,000 deaths per year [1]–[2]. In China, gastric cancer was predicted to be the third most common cancer in 2005 with 0.4 million new cases and 0.3 million deaths reported [3]. The treatment of gastric cancer includes a combination of surgery, chemotherapy, and radiation therapy. But nearly 60% of affected patients succumb to gastric cancer after a curative resection alone or after a curative resection with subsequent adjuvant therapy [4]. Gastric cancer is a heterogeneous disease in both histology and genetics; hence, patient outcome is difficult to predict using classic histological classifications. Gastric carcinogenesis is a multifactorial and multistep process that involves activating oncogenes and inactivating tumor suppressor genes in different stages of gastric cancer progression. Recently, several new oncogenes and tumor suppressor genes associated with gastric cancer have been identified. Therefore, it is clinically important to find efficient new targets for the early diagnosis and effective treatment of gastric cancer.

AZGP1 (2-zinc-glycoprotein 1, Zn-alpha 2-glycoprotein) is a 41 kDa soluble protein with a major histocompatibility complex-1 (MHC-1)-like fold in its structure, and it was initially identified and purified in human serum in 1961 [5]. The gene for AZGP1, assigned to the chromosome 7q22.1 through fluorescent hybridization karyotyping, is comprised of four exons and three introns [6], [7]. Using immunohistochemical studies, it has been found that AZGP1 is expressed mainly in epithelial cells of the breast, the prostate, the liver and various other gastrointestinal organs [8]. In line with its production by secretory epithelial cells, AZGP1 is found in a number of body fluids [9], [10], [11].

AZGP1 is a multidisciplinary protein that participates in many important functions in the human body, including fertilization [11], immunoregulation [12] and lipid mobilization [13], [14], [15]. AZGP1 is also associated with cancer cachexia. AZGP1 has a high level of amino-acid sequence homology with tumor-derived lipid-mobilizing factor [14], and in a mouse model of AZGP1-producing tumors, AZGP1 stimulated lipolysis in adipocytes leading to cachexia [16]. Recently, it has been shown that AZGP1 is also involved in carcinogenesis and tumor differentiation. Protein and mRNA expression assays have shown a relationship between the AZGP1 levels and the histologic grade of breast cancer tumors [17], [18]. Moreover, many studies suggest that AZGP1 is a potential serum marker of prostate cancer [9], [19]. In addition, it has been shown that AZGP1 acts as a novel tumor suppressor in pancreatic cancer [20]. However, so far the expression status of AZGP1 and prognostic value of this protein in primary gastric cancers have not been reported.

In this study, we analyzed the AZGP1 expression level in gastric cancers by using real-time quantitative RT-PCR (qRT-PCR), western blotting and immunohistochemistry. Furthermore, we identified the relationship between AZGP1 expression and the clinicopathological features of gastric cancer, and we evaluated the prognostic value of AZGP1 expression for the post-resection survival of gastric cancer patients.

## Results

### AZGP1 mRNA Expression Analyzed with qRT-PCR

**Figure 1:**
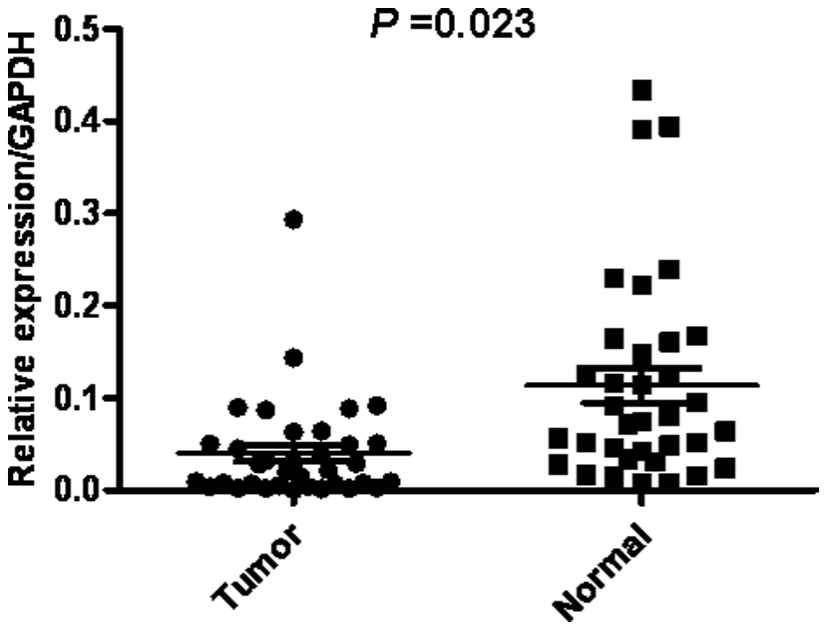
qRT-PCR analysis of AZGP1 expression in gastric cancer patients. Relative expression of AZGP1 in gastric cancer tumor tissues compared to adjacent non-tumor tissues (n = 35) assessed by qRT-PCR (*P* = 0.023).

The transcriptional levels of AZGP1 were determined with qRT-PCR assays using 35 pairs of resected specimens (tumor tissue samples and matched adjacent non-tumor tissue samples) from gastric cancer patients. The AZGP1 mRNA levels were significantly reduced in 28 (80%) tumor tissue samples compared with the matched adjacent non-tumor tissue samples (*P* = 0.023, Figure 1).

### AZGP1 Expression Analyzed by Western Blotting

**Figure 2:**
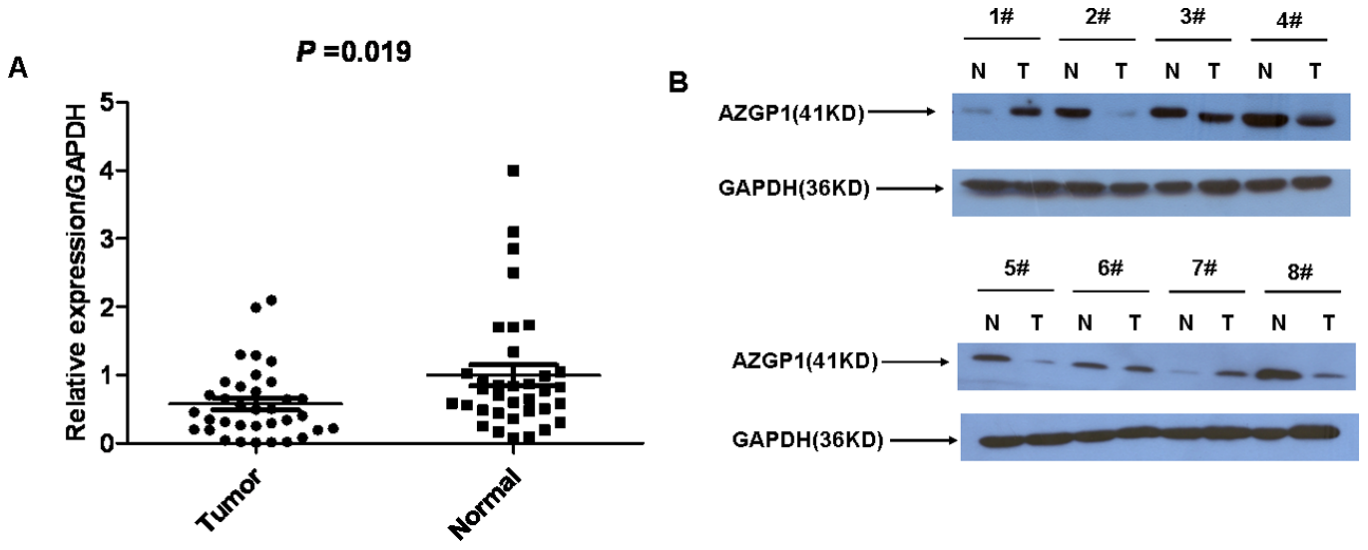
Western blotting analysis of AZGP1 expression in gastric cancer patients. (A) The relative AZGP1 protein expression levels was significantly decreased in gastric cancer tissues compared with the matched adjacent non-tumorous tissues (n = 35, *P* = 0.019); (B) Representative results of western blotting analysis of AZGP1 protein expression in eight gastric cancer tissues (T) and the matched adjacent non-tumorous tissues (N).

The AZGP1 protein levels in the resected gastric cancer samples were determined by western blotting. The results showed a band for AZGP1 at 41 kDa, and the amount of AZGP1 protein present was measured by densitometry and height. Consistent with the qRT-PCR results, a decrease in AZGP1 expression was observed in 25 (71.4%) of the gastric tumor tissues compared with the matched adjacent non-tumor tissues (*P* = 0.019, Figure 2A). Eight pairs of representative gastric tumor tissues and the matched adjacent non-tumor tissues were shown in Figure 2B.

### Immunohistochemical Analysis of AZGP1 Expression in Gastric Cancer Tissue Samples and its Relationship with the Clinicopathological Features

**Figure 3:**
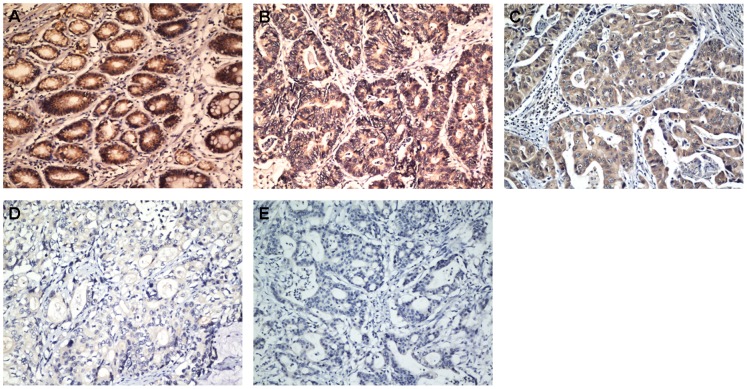
Immunohistochemical detection of the AZGP1 protein expression in gastric cancer and surrounding non-tumor tissues. (A) Normal gastric tissues, scored as AZGP1 (+++);(B) gastric cancer, scored as AZGP1 (+++); (C) gastric cancer, scored as AZGP1 (++);(D) gastric cancer, scored as AZGP1 (+); and (E) gastric cancer, scored as AZGP1 (-). Original magnification: A - D × 200.

In order to confirm the molecular biological findings and investigate the clinicopathological the prognostic roles of AZGP1 expression, we performed immunohistochemical analysis in 248 paraffin-embedded gastric cancer sections. The positive expression of AZGP1 was localized to the cytoplasm(Figure S1). Among the 248 gastric cancer samples, 117 (47.2%) showed high AZGP1 expression (AZGP1++ or AZGP1+++), whereas the remaining 131 cases (52.8%) displayed low AZGP1 expression (AZGP1- or AZGP1+) (Figure 3, Table 1). Normal gastric tissues showed the strongest AZGP1 positive staining (Figure 3A).

**Table 1:** Relationship between AZGP1 expression and clinicopathologic features of patients with gastric cancer.

Variables	Number	AZGP1 expression		*P*-value
		Low	High	
**Age (years)**				0.788
<60	140	75	65	
≥60	108	56	52	
**Gender**				0.586
Male	161	83	78	
Female	87	48	39	
**Tumor size (cm)**				0.143
≤5.0	160	79	81	
>5.0	88	52	36	
**Histological grade**				0.005*
Well/Moderately differentiated (G1/G2)	94	39	55	
Poorly differentiated (G3)	154	92	62	
**Tumor location**				0.011*
Distant	139	62	77	
Proximal	95	59	36	
Total	14	10	4	
**Radical resection**				0.175
Yes	222	114	108	
No	26	17	9	
**Tumor invasion (T)**				0.008*
T1/T2/T3	93	39	54	
T4a/T4b	155	92	63	
**Nodal status (N)**				0.469
No	69	39	30	
Yes	179	92	87	
**Metastasis status (M)**				0.287
M0	226	117	109	
M1	22	14	8	

* Statistically significant (*P*<0.05).

Based on the categories that we defined in the afore mentioned methods, the data showed that the low expression of AZGP1 was significantly correlated with tumor location (*P* = 0.011), histological grade (*P* = 0.005) and T stage (*P* = 0.008), but not with age, gender, tumor size, radical resection, nodal status (N stage) or metastasis status (M stage). The micrographs are shown in Figure 3.

### Correlation between AZGP1 Expression Based on Immunohistochemistry and Patient Survival

**Figure 4:**
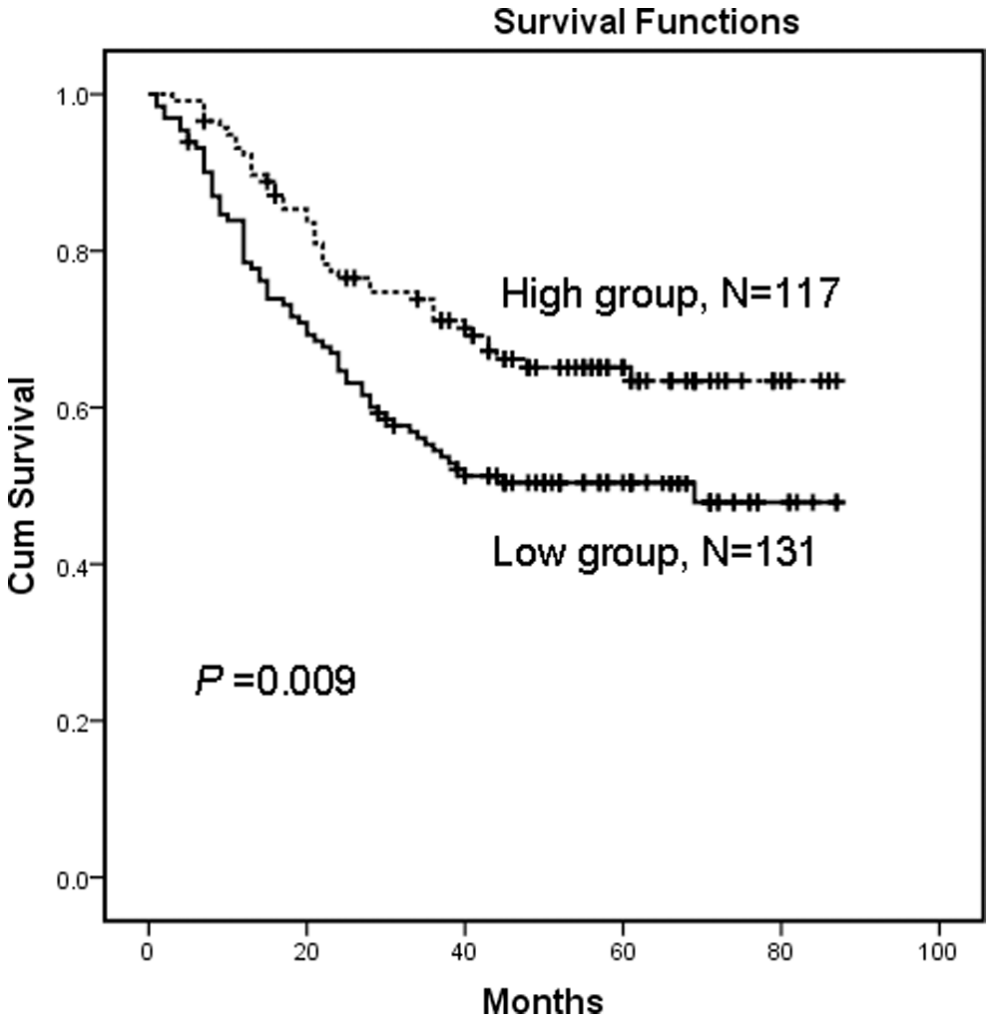
Kaplan–Meier survival curves of gastric cancer patients (n = 248) after surgical resection. Low expression of AZGP1 correlated with poor patient survival. Patients in the high AZGP1 expression group exhibited significantly better survival than the low AZGP1 expression group (log-rank test: *P = *0.009).

The median survival time of the 248 gastric cancer patients was 45 months (range 2–89 months). The overall survival rate and 5-year survival rate were significantly improved in high AZGP1 expression group than the low expression group [64.2% vs. 49.5% (overall survival rate) and 65.1% vs. 50.4% (5-year survival rate), respectively, *P* = 0.009, Figure 4].

### Univariate and Multivariate Analyses

Univariate and multivariate analyses were performed to compare the impact of AZGP1 expression and other clinicopathological parameters on prognosis. Based on a univariate analysis that included all 248 patients, 8 factors were found to have statistically significant associations with overall survival. This analysis took following factors in consideration: tumor location, tumor size, histological grade, AZGP1 expression levels, TNM stage (7th edition TNM classification) and whether or not a radical resection was performed (Table 2). All 8 factors were included in a multivariate Cox proportional hazards model to adjust for the effects of the covariates. Based on this model, the tumor location, AZGP1 expression levels, T stage and N stage of the tumor were confirmed as independent prognostic factors (Table 2).

**Table 2:** Univariate and multivariate survival analysis of clinic-pathologic variables in 248 cases of gastric carcinoma patients.

Variables	Univariate analyses			Multivariate analyses		
	HR	(95% CI)	*P*-value	HR	(95% CI)	*P*-value
**Gender** (female vs. male)	1.150	0.789–1.676	0.468			
**Age** (years) (≥60 vs. <60 )	1.231	0.855–1.773	0.264			
**Location** (total/proximal/distal)	2.146	1.447–3.175	0.015*	1.527	1.185–2.151	<0.001*
**Size** (cm) (>5 vs. ≤5)	2.029	1.407–2.924	<0.001*	1.376	0.918–2.064	0.123
**Histological grade** (G3/G2/G1)	1.376	1.060–2.786	0.017*	1.306	0.843–2.024	0.232
**Radical resection** (No vs. Yes)	5.130	3.266–8.057	<0.001*	2.916	1.450–6.165	0.379
**AZGP1** (low vs. high)	1.748	1.338–3.265	0.010*	1.681	1.134–2.494	0.011*
**T stage** (T4b+T4a/T3+T2+T1)	3.932	2.425–6.377	<0.001*	1.985	1.150–3.427	0.014*
**N stage** (Yes/No)	4.032	2.106–7.718	<0.001*	3.776	2.191–6.510	<0.001*
**M stage** (M1 vs. M0)	7.047	4.378–11.343	<0.001*	2.548	0.572–9.354	0.220

HR, hazard ratio; CI, confidence interval;

* Statistically significant (*P*<0.05).

## Discussion

Gastric cancer remains one of the most deadly human malignancies. Even with advances in diagnosis and therapy, the prognosis for gastric cancer is still dismal [1], [21]. The clinical outcome of gastric cancer depends on a series of tumor characteristics, such as tumor growth, differentiation, invasion and distant metastasis, which are regulated by a variety of related genes. Therefore, it is generally considered that genetic alterations leading to the activation of oncogenes and the inactivation of tumor suppressors are the underlying causes of cancer pathogenesis. The sequential gain of oncogenes and loss of tumor suppressors provide the necessary foundation for the step-wise progression of solid tumors from initiation to transformation and tumor progression [22], [23], [24]. Previously, AZGP1 has been reported to possess tumor suppressive properties in breast cancer, prostate cancer, pancreatic cancer and some other malignant tumors [17], [20], [25]; however the role of AZGP1 in primary gastric cancer has not yet been evaluated. Our study indicated that AZGP1 expression was significantly reduced at both the mRNA and protein levels in tumor tissue samples, compared with expression in matched adjacent non-tumor tissue samples. Consistent with our study, Brysk MM et al. also demonstrated that the AZGP1 levels are higher in normal oral tissues than in oral tumors [26]. Gagnon S et al. also proved that AZGP1 was present in benign hyperplastic glands in 91.1% of cases but in only 40.7% (poorly differentiated component) to 48.5% (well differentiated component) of prostatic adenocarcinomas and only 8% of metastases [27]. These study results support the hypothesis that AZGP1 may serve as a tumor suppressor in some cancers.

To further validate this reduction of AZGP1 expression in primary gastric cancer, we performed immunohistochemical analysis with a rabbit anti-hAZGP1 antibody. We observed lower expression of AZGP1 immunostaining in proximal or total gastric cancer compared with distant gastric cancer tissues. Some studies have proven that proximal gastric cancer patients have a worse survival than distant gastric cancer patients [28]. These data suggested that the low expression of AZGP1 in gastric cancer is associated with more malignant phenotypes. In addition, we discovered that decreased expression of AZGP1 was associated with poorly differentiated adenocarcinomas (G3 vs. G1/G2), indicating that AZGP1 may induce the differentiation of gastric cancer. These results are consistent with the findings of Diez et al. [17], [18] who described an association between high AZGP1 expression and high levels of differentiation in breast cancer. Similar findings have also been reported for prostate cancer [19], breast cancer and pancreatic cancer [26]. Furthermore, we detected that the low expression of AZGP1 was associated with advanced T stage. These results implied that the low expression of AZGP1 might promote tumor growth. In accordance with our study, Irmak S et al. suggested that AZGP1 is related to the development of superficial bladder cancer and its transformation to an invasive phenotype [29]. These findings collectively indicate an important role for AZGP1 in the differentiation and growth of gastric cancer.

Using the Kaplan–Meier survival analysis, patients in our study with low AZGP1 expression had a significantly shorter overall survival than those with high expression levels. Univariate analyses showed that the decreased expression of AZGP1 in gastric cancer tissues was significantly associated with the overall survival rate and the 5-year survival rate. Multivariate analysis demonstrated that AZGP1 expression, together with some traditional prognostic factors such as tumor location, T stage and N stage, were independent risk factors in the prognosis of gastric cancer patients. These results suggested that AZGP1 might serve as a new predictor of prognosis in gastric cancer patients after surgical resection.

The molecular mechanisms of AZGP1’s tumor suppressive properties are still unclear. AZGP1 belongs to the macroglobulin family, an ancient and evolutionarily conservative link of the immune system. Zorin NA et al. suggested that the capacity of macroglobulins for binding hydrolases makes the inhibition of enzyme-mediated tumor invasion possible [30]. At the same time, an excess of macroglobulin/hydrolase complexes can activate apoptosis [31]. He N et al. reported that AZGP1 also down-regulates cyclin-dependent kinase, which is responsible for regulating the G2-M transition, a rate-limiting step in the cell cycle. This suggests that AZGP1 indirectly plays a role in hindering tumor progression [32]. AZGP1 was proposed as a tumor suppressor in pancreatic cancer by Kong B. et al. Their study suggested that the AZGP1 gene induces mesenchymal-to-epithelial transdifferentiation by inhibiting TGF-b-mediated ERK signaling [20]. The functional role and mechanisms of AZGP1 in gastric cancer need further investigation.

In conclusion, we first investigated the expression levels and prognostic value of AZGP1 in primary gastric cancers in this study. Our study results suggested that AZGP1 might serve as a candidate tumor suppressor and prognostic biomarker in primary gastric cancers and be a potential target for therapeutic intervention; however, the molecular mechanisms involved in the regulation of AZGP1 in gastric cancer warrants further investigation.

## Materials and Methods

### Ethics Statement

This research was approved by the Ethics Committee of Sun Yat-sen University Cancer Center, and written informed consent was obtained from each patient involved in the study.

### Human Tissue Sample*s*

A total of 35 paired cancerous and matched adjacent noncancerous gastric mucosa tissues were collected from gastric cancer patients undergoing gastrectomy at Sun Yat-sen University Cancer Center from 2010 to 2011. After surgical resection, the fresh tissues were immediately immersed in RNAlater (Ambion, Inc., USA) to avoid RNA degradation, stored at 4°C overnight to allow thorough penetration of RNAlater into the tissue and then frozen at −80°C until the RNA and protein extraction was performed. Another 248 paraffin-embedded primary gastric carcinoma samples that had been collected between 2005 and 2007 were obtained from the Sun Yat-sen University Cancer Center. None of these patients had received radiotherapy or chemotherapy prior to surgery. The histopathological type and stage of the gastric cancer were determined according to the criteria of the World Health Organization classification and the TNM stage set out by the Union for International Cancer Control.

### Extraction of Total RNA and Real-time Quantitative RT-PCR

Total RNA was extracted using TRIzol (Invitrogen, Carlsbad, California, USA) according to the manufacturer’s protocol. The total RNA concentration was assessed by measuring absorbance at 260 nm using a NANO DROP spectrophotometer (ND-1000, Thermo Scientific, USA). Reverse transcription (RT) to synthesize the first-strand of cDNA was performed using 2 µg of total RNA treated with M-MLV reverse transcriptase (Promega, USA) according to the manufacturer’s recommendations. The resulting cDNA was then subjected to real-time quantitative RT-PCR for evaluation of the relative mRNA levels of AZGP1 and GAPDH (glyceraldehyde-3-phosphate dehydrogenase, as an internal control) with the following primers: AZGP1 forward: 5′-GGAAGCAGGACAGCCAACTT-3′, and reverse: 5′-TTATTCTCGATCTCACAACCAAAC-3′; GAPDH forward: 5′-CTCCTCCTGTTCGACAGTCAGC-3′, and reverse: 5′-CCCAATACGACCAAATCCGTT-3′. Gene-specific amplification was performed using an ABI 7900HT real-time PCR system (Life Technologies, Carlsbad, California, USA) with a 15-µl PCR mix containing 0.5 µl of cDNA, 7.5 µl of 2× SYBR Green master mix (Invitrogen, Carlsbad, California, USA), and 200 nM of the appropriate oligonucleotide primers. The mix was preheated at 95°C (10 min) and then amplified at 95°C (30 sec) and 60°C (1 min) for 45 cycles. The resolution curve was measured at 95°C for 15 sec, 60°C for 15 sec and 95°C for 15 sec. The Ct (threshold cycle) value of each sample was calculated from the threshold cycles with the instrument’s software (SDS 2.3), and the relative expression of AZGP1 mRNA was normalized to the GAPDH value. The data were analyzed using the comparative threshold cycle (2^−ΔCt^) method as the following formula: Relative expression level = 2^−ΔCt^ = 2^−Ct (GAPDH)^ - 2^−Ct (AZGP1)^, in which Ct(GAPDH) means the Ct value of GAPDH and Ct(AZGP1) means the Ct value of AZGP1.

### Western Blotting Analysis

The homogenized gastric cancer samples, including tumor and nontumor tissues, were lysed in RIPA lysis buffer, and the lysates were harvested by centrifugation (12,000 rpm) at 4°C for 30 min. Approximately 20 µg protein samples were then separated by electrophoresis in a 12% sodium dodecyl sulfate polyacrylamide gel and transferred onto a polyvinylidene fluoride membranes. After blocking the non-specific binding sites for 60 min with 5% non-fat milk, the membranes were incubated overnight at 4°C with a rabbit monoclonal antibody against AZGP1 (PTG Company, USA, at a 1∶200 dilution). The membranes were then washed three times with TBST (tris-buffered saline with tween-20) for 10 min and probed with the horseradish peroxidase (HRP)-conjugated goat anti-rabbit IgG antibody (Immunology Consultants Laboratory, USA, at a 1∶2000 dilution) at 37°C for 1 hour. After three washes, the membranes were developed by an enhanced chemiluminescence system (Cell Signaling Technology, Danvers, Massachusetts, USA). The band intensity was measured by densitometry using the Quantity One software (Bio-Rad Laboratories, Inc. Hercules, CA, USA). The protein levels were normalized to that of GAPDH detected using a mouse anti-human GAPDH monoclonal antibody (Shanghai Kangchen, China, at a 1∶10000 dilution).

### Immunohistochemistry Analysis

The tissue sections were deparaffinized with dimethylbenzene and rehydrated through 100%, 95%, 90%, 80% and 70% ethanol. After three washes in PBS (phosphate-buffered saline), the slides were boiled in antigen retrieval buffer containing 0.01 M sodium citrate-hydrochloric acid (pH = 6.0) for 15 min in a microwave oven. After rinsing with PBS, the tissue sections were incubated with the primary antibody and the slides were then rinsed in 3% peroxidase quenching solution (Invitrogen) to block endogenous peroxidase. The sections were then incubated with a rabbit monoclonal antibody against AZGP1 (PTG Company, USA, at a 1∶200 dilution) at 4°C overnight and then incubated with horseradish peroxidase (HRP) (ChemMateTM DAKO EnVisionTM Detection Kit) at room temperature for 30 min. After washing in PBS, the visualization signal was developed with 3, 3′-diaminobenzidine (DAB) solution, and all of the slides were counterstained with hematoxylin. As negative controls, adjacent sections were processed as described above except that they were incubated overnight at 4°C in blocking solution without the primary antibody.

The specimens were analyzed by three observers (Chunyu Huang, Lin Lv, and Jingjing Zhao) who were blinded to the patients’ clinical outcomes. Discrepancies between the observers were found in less than 10% of the examined slides, and a consensus was reached after further review. The total AZGP1 immunostaining score was calculated as the sum of the percent positivity (the percentage of the positively stained tumor cells) and the staining intensity. The percent positivity was scored as “0” (<5%, negative), “1” (5–25%, sporadic), “2” (25–50%, focal), or “3” (>50%, diffuse). The staining intensity was scored as “0” (no staining), “1” (weakly stained), “2” (moderately stained), or “3” (strongly stained). The total AZGP1 immunostaining score ranged from 0 to 9. We defined the AZGP1 expression levels as follows: “−” for a score of 0–1, “+” for a score of 2–3, which were defined as low expression; “++” for a score of 4–6, and “+++” for a score >6, which were defined as high expression.

### Follow-Up

The postoperative follow-up was conducted at our outpatient department and included clinical and laboratory examinations every 3 months for the first 2 years, every 6 months during the third to fifth years, and annually for an additional 5 years or until patient death, whichever occurred first. Overall survival, which was defined as the time from the operation to the patient’s death or the last follow-up, was used as a measure of prognosis. There were about 4% patients lost follow-up.

### Statistical Analysis

A paired-samples t-test was used to compare the AZGP1 mRNA levels in the tumor tissue samples and the adjacent non-tumor tissue samples. The *χ* ^2^ test for proportion and Pearson’s correlation coefficients were used to analyze the relationship between AZGP1 expression and various clinicopathological characteristics. Overall survival curves were calculated with the Kaplan-Meier method and were analyzed with the log-rank test. Cox proportional-hazard analysis was used for univariate and multivariate analysis to explore the effect of clinicopathological variables and AZGP1 expression on survival. A two-sided *P*-value <0.05 was considered to be statistically significant. All statistical analyses were performed with SPSS software (version 17.0; SPSS Inc., Chicago, IL, USA).

## Supporting Information

Figure S1 **Immunohistochemical detection of the AZGP1 protein expression in gastric cancer tissue.** The positive expression of AZGP1 was localized to the cytoplasm.
(TIF)
