# Supplementary figures and images for: Decreased Expression of AZGP1 Is Associated with Poor Prognosis in Primary Gastric Cancer
Source: PLoS One. 2013 Jul 23;8(7):e69155. doi: 10.1371/journal.pone.0069155 (PMC3720544; doi:10.1371/journal.pone.0069155)

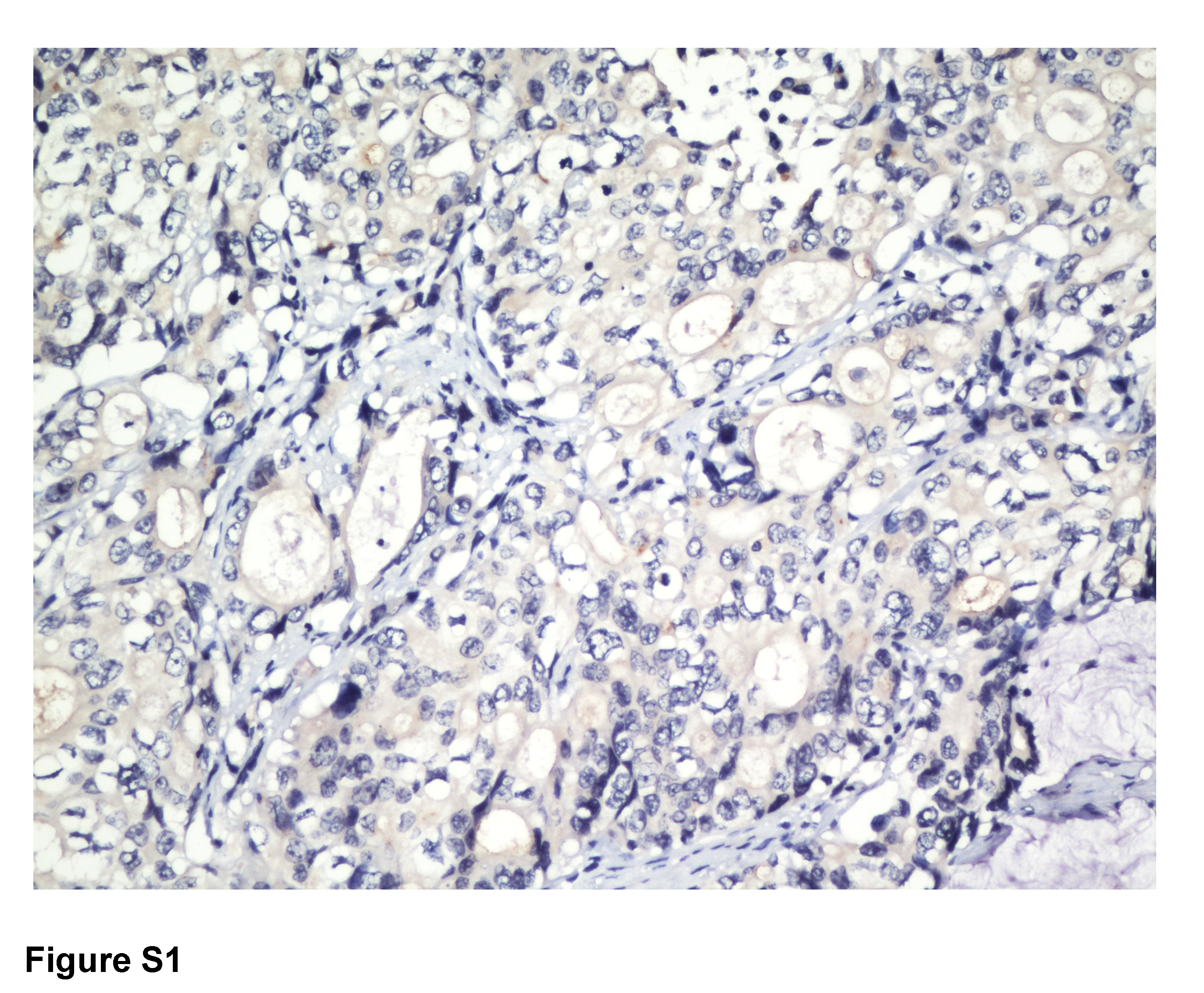

Supplement: Figure S1 — Immunohistochemical detection of the AZGP1 protein expression in gastric cancer tissue. The positive expression of AZGP1 was localized to the cytoplasm. (TIF) [file pone.0069155.s001.tif]
